# Community-based accompaniment for adolescents transitioning to adult HIV care in urban Peru: a pilot study

**DOI:** 10.1007/s10461-022-03725-2

**Published:** 2022-07-05

**Authors:** Valentina Vargas, Milagros Wong, Carly A. Rodriguez, Hugo Sanchez, Jerome Galea, Alicia Ramos, Liz Senador, Lenka Kolevic, Eduardo Matos, Eduardo Sanchez, Renato A. Errea, Karen Ramos, Catherine Beckhorn, Andrew Lindeborg, Carlos Benites, Leonid Lecca, Sonya Shin, Molly F. Franke

**Affiliations:** 1 Department of Global Health and Population, Harvard T.H. Chan School of Public Health, Boston, USA; 2 Socios En Salud Sucursal Peru, Lima, Peru; 3 Department of Global Health and Social Medicine, Harvard Medical School, Boston, USA; 4 Epicentro, Lima, Peru; 5 School of Social Work, University of South Florida, Florida, USA; 6 College of Public Health, University of South Florida, Florida, USA; 7 Servicio de Infectología, Instituto Nacional del Salud del Niño, Lima, Peru; 8 Programa de ITS, VIH/SIDA y hepatitis, Ministerio de Salud, Lima, Peru; 9 Servicio de Infectología, Hospital Nacional Arzobispo Loayza, Lima, Peru; 10 Servicio de Enfermedades Infecciosas y Tropicales, Hospital Nacional Hipólito Unanue, Lima, Peru; 11 Division of Global Health Equity, Brigham and Women’s Hospital, Boston, USA

**Keywords:** Adolescents, Social support, Adherence, Differentiated care, HIV care continuum

## Abstract

We piloted a community-based intervention to improve outcomes among adolescents living with HIV who were transitioning to adult-oriented care in Lima, Peru. We assessed feasibility and potential effectiveness, including within-person changes in self-reported adherence, psychosocial metrics (NIH Toolbox), and transition readiness (“Am I on TRAC” questionnaire, “Got Transition” checklist). From October 2019 to January 2020, we enrolled 30 adolescents (15–21 years). The nine-month intervention consisted of logistical, adherence and social support delivered by entry-level health workers and group sessions to improve health-related knowledge and skills and social support. In transition readiness, we observed within-person improvements relative to baseline. We also observed strong evidence of improvements in adherence, social support, self-efficacy, and stress, which were generally sustained three months post-intervention. All participants remained in treatment after 12 months. The intervention was feasible and potentially effective for bridging the transition to adult HIV care. A large-scale evaluation, including biological endpoints, is warranted.

**Supplementary information:**

The online version contains supplementary material available at 10.1007/s10461-022-03725-2.

## Resumen

Piloteamos una intervención comunitaria para mejorar los resultados de adolescentes viviendo con el VIH que se encontraban en transición a la atención de VIH orientada a adultos en Lima, Perú. Evaluamos la viabilidad y la eficacia potencial, incluidos los cambios personales en la adherencia auto-reportada, criterios psicosociales (NIH Toolbox), y preparación para la transición (cuestionario “Estoy en el TRAC”, lista de verificación “Got Transition”). Desde octubre de 2019 hasta enero de 2020, enrolamos a 30 adolescentes (15 a 21 años). La intervención de nueve meses consistió en apoyo logístico, de adherencia y social brindado por técnicos de enfermería y sesiones grupales para mejorar tanto el conocimiento y las habilidades relacionados con la salud, como el apoyo social. En cuanto a la preparación para la transición, observamos mejoras comparada con el basal. También observamos una fuerte evidencia de mejoras en la adherencia, apoyo social, autoeficacia y estrés, que se mantuvieron, generalmente, tres meses después del fin de la intervención. Todos los participantes seguían en tratamiento después de 12 meses. La intervención fue factible y potencialmente efectiva para tender un puente en la transición a la atención del VIH para adultos. Esto amerita una evaluación a mayor escala y que incluya criterios clínicos.

## Introduction

Expanded access to antiretroviral therapy (ART) has led to major improvements in the survival of children who acquire HIV early in life. This means that providers and programs are faced with the challenge of caring for a group of patients living with HIV that is also dealing with the many developmental, emotional and behavioral issues of adolescence. These include the desire for peer acceptance, vulnerability to mental health and substance use disorders, and the potential consequences of early sexual debut (e.g., pregnancy, HIV transmission, other sexually-transmitted infections) [1–6]. And, adolescents living with HIV (ALWH) may lack the coping strategies and support needed to effectively manage common HIV-related challenges, such as diagnosis disclosure, HIV-related stigma and social isolation, neurocognitive delays, and navigating romantic relationships [1–3, 7–10]. In spite of their unique needs, ALWH have rarely been targeted for intervention studies to improve health outcomes [11–13]. The absence of tailored interventions for adolescents may in part explain their disproportionately high rates of attrition to care relative to other age groups [14, 15] and suboptimal rates of viral suppression and medication adherence during adolescence [16–18].

For many ALWH, the transition to adult HIV care is a tumultuous time, marked by episodic care and declines in HIV viral load suppression and CD4 cell counts [19, 20]. This transition, which often occurs in late adolescence, can be accompanied by logistical and emotional challenges, as adolescents find themselves newly independent with different medical providers in an unfamiliar and often less supportive environment [21, 22]. The transition may be particularly challenging for ALWH who are not adequately prepared, with important gaps in life skills and HIV-related knowledge.[23, 24] These deficits, along with a low level of caregiver or health provider support, differences in care cultures between pediatric and adult clinics, out-of-pocket expenses, and challenges related to transportation and health insurance, contribute to unsuccessful transitions to adult care.[10, 25–29] Although retention rates post-transition are heterogenous across settings, they tend to fall well below 95-95-95 targets, and adolescents with evidence of disease progression, unsuppressed viral load, or unstable adherence prior to transition are at particularly high risk of poor outcomes in the post-transition period.[30, 31] Although several reviews suggest good transition outcomes may be achieved with individualized transition plans that address the multifaceted needs of adolescence [10, 22, 32–34], there are few rigorous studies testing promising interventions [35, 36] and a notable lack of evidence on which interventions might be most effective in supporting ALWH through this period.

Alongside an urgent need to identify effective transition interventions is a growing recognition of the need for interventions that are tailored to local context and to specific subgroups. A recent review on care transition studies for ALWH identified an absence of studies from Latin America and the Caribbean, where the experiences of ALWH likely differ from those in high-income or high prevalence settings [36]. There is also a stark need for transition studies among adolescents with behaviorally-acquired HIV[10], including young men who have sex with men (MSM) and transgender women, as their transition experiences may differ from those of adolescents living with perinatal HIV infection [37]. As a first step toward addressing these research gaps, we conducted a pilot study to examine whether an intervention for ALWH, rooted in community-based support by an entry-level health worker, could be a feasible approach for bridging the transition to adult HIV care among a diverse group of adolescents in the urban Latin American setting of Lima, Peru.

## Methods

### Study setting

In Peru, there are an estimated 91,000 people living with HIV, with the highest concentration in the capital, Lima [38]. While perinatal infection constitutes the majority of HIV infections in younger adolescents, Peru’s HIV epidemic has remained concentrated [39] among MSM and transgender women, with one in ten and one in three thought to be living with HIV. Between 2017 and September 2021, there were 8951 new cases of HIV diagnosed among youth between the ages of 15 and 24 years in Peru, and 79% of these (n = 7041) occurred in men [40].

ART was made available free-of-charge through the public sector in 2004 and remains largely centralized with most patients receiving treatment through public sector hospitals. Care under Peru’s National HIV Program is provided by a multidisciplinary team of providers including physicians, nurses, psychologists, and peer-supporters. ART is typically dispensed monthly, but may be dispensed at longer intervals for patients who have established consistent adherence.

For adolescents that acquired HIV in early childhood, the transition from a pediatric to an adult HIV clinic often occurs at age 18, though it may occur as young as 15 for adolescents transitioning within the same health facility. Older, newly-diagnosed adolescents may transition directly into adult HIV care, bypassing the pediatric clinic. Pregnancy during adolescence is common with 17% of 20- to 24-year-old women giving birth before age 18 [41]. If an adolescent in pediatric care becomes pregnant, she immediately transitions to an adult HIV clinic, regardless of age. The existing standard of care for adolescents transitioning to adult care includes a public health insurance referral and a series of consults with a nurse, infectious disease specialist, psychologist, and social worker prior to transition. The patient is given a report from each provider team, which the ALWH is responsible for bringing to their first adult clinic visit. Administrative requisites for transition often vary by individual but may include registration for public health insurance, procurement of a national ID card (e.g., for foreign migrants), or securing a health center transfer (i.e., due to housing relocation).

While, we know of no published studies examining health outcomes among ALWH following transition to adult care in Peru, studies suggest that adherence, CD4 cell count, and viral suppression decrease throughout adolescence [17], with lack of positive caregiver support a major barrier to treatment adherence [42].

### Participants

We included ALWH aged 15 to 21 years who were on or newly initiating ART, enrolled in HIV care at a participating public sector clinic, and scheduled to transition to adult HIV care, either due to a recent HIV diagnosis or because they had aged out of their pediatric clinic. We consecutively enrolled patients representing the three common transition experiences in this setting, aiming for approximately ten participants per group: adolescents transitioning from a pediatric clinic to an adult clinic at a new facility; adolescents transitioning from a pediatric to an adult clinic within the same facility; and adolescents newly initiating treatment in an adult facility. ALWH who were previously lost from care during transition but interested in re-engaging in treatment were also eligible to participate. Adolescents were excluded if they lived outside of metropolitan Lima. Enrollment took place from October 2019 to January 2020 at three public sector hospitals (one pediatric facility and two facilities with distinct on-site pediatric and adult clinics). Ministry of Health clinical collaborators consecutively referred eligible participants to study staff. A Youth Advisory Board comprising a diverse group of young people living with HIV provided feedback on all aspects of the study. Adolescents who had previously withdrawn from care were referred by their clinicians or Youth Advisory Board members and contacted by study staff.

### Study intervention

The intervention was nicknamed “PASEO” (meaning ‘crossing’ or ‘passage’ in Spanish) after its core elements: peer engagement, accompaniment, support and education. In short, PASEO aimed to provide in-person accompaniment to ALWH throughout the transition process through the provision of individualized support, including adherence support, to address barriers to care and treatment (e.g., transport costs, inaccessibility of services, lack of caregiver support) by providing medical, material, and social support delivered by compensated entry-level or lay health workers who liaise with formal health services [43].

PASEO also aimed to foster health-related skills, knowledge, and psychosocial well-being needed to thrive in the adult clinic and more broadly. Intervention activities were delivered in two phases, an intensive phase and a taper phase (see Table, Electronic Supplementary Materials 1, which describes the components of the intervention). The intensive phase began just prior to transition (defined as the last date of care at the pediatric clinic or, for adolescents initiating treatment directly in an adult clinic, the ART initiation date) and ended after six months. A three-month taper phase, during which the intensity of study activities was reduced, followed the intensive phase. Below, we describe each intervention component in detail as well as adaptations made to accommodate stay-at-home orders invoked on March 15, 2020, due to the SARS-CoV-2 pandemic. Participants had received between 1.4 and 5.3 months of in-person intervention (median: 3.1 [interquartile range (IQR): 2.2, 4.2]) when in-person activities were suspended and programming was implemented virtually. Adolescents were provided phone credit to enable participation in virtual programming.

*Health system navigation and clinic accompaniment.* To facilitate the transition to care in the adult clinic, trained health promoters (entry-level health workers with a technical degree) employed by the nongovernmental organization Socios En Salud accompanied adolescents to their first appointments; facilitated enrollment in public health insurance and completion of other administrative requisites; helped troubleshoot logistical and/or social challenges (e.g., housing instability); and served as a liaison between the patient and pediatric and adult clinical providers. Clinic visit transportation was provided based on economic need, as determined by the study social worker. All but one participant had attended at least one accompanied in-person clinic visit prior to SARS-CoV-2 stay-at-home orders. The exception was an individual who was scheduled to re-initiate care in mid-March 2020. While hospital guidelines precluded in-person accompaniment for this participant, intervention staff coordinated with HIV program staff to ensure that adolescent would be seen in the clinic. Study staff coordinated private telehealth appointments for adolescents requiring clinical consultation during stay-at-home orders.

*Routine check-in visits.* Health promoters visited the participant’s home or another mutually-agreed upon location at least monthly to review ART adherence, identify barriers to care and adherence, remind patients of upcoming medical encounters, screen for and follow-up on clinical and social problems and offer social support. The frequency of check-ins was individualized and commensurate with the level of support needed. Visits tended to occur more frequently for participants lacking family support, re-initiating ART or with a history of unstable adherence.

*Directly Observed Treatment (DOT).* Home-based DOT was offered to adolescents identified as at risk of non-adherence (based on pediatric health provider assessment or a detectable viral load at one of two previous measurements). ALWH newly initiating ART chose whether they wished to receive DOT. Eligible ALWH who opted into DOT were assigned to a Ministry of Health community health worker from the same neighborhood to observe ART ingestion daily, ensure treatment was taken as prescribed and offer social support. Following invocation of stay-at-home orders, DOT was conducted virtually by Socios En Salud health promotors via telephone, video or short message service, depending on participant preference. Some participants elected to participate in group video DOT, creating an additional opportunity to build social support.

*Social support groups, health education sessions and skill-building workshops*. Social support groups took place at least once monthly (10 adolescents per group). The two-hour sessions were facilitated by a Peruvian study psychologist and one peer youth living with HIV. Based on timing of enrollment, adolescents were eligible to have attended between two and five in-person sessions prior to stay-at-home orders. Conducting these sessions online was largely unsuccessful: many adolescents felt they lacked privacy at home to discuss personal topics [44], and others could not use video due to unstable internet. To build and sustain social connectedness, staff implemented online social activities including an HIV-related meme competition, a TikTok video competition, and a virtual talent show.

Health education sessions and skill-building workshops were conducted on topics such as HIV, substance use, sexual health, self-esteem, nutrition, and gender and sexual identity. In-person sessions took place at least once monthly (coinciding with social support group sessions), while virtual sessions took place one to three times weekly (total of 41). For virtual sessions, participants could join live, allowing interaction with the speaker and other participants via chat, or view a recording.

*Mental health screening, referral and support.* Participants were evaluated for depressive symptoms using the Patient Health Questionnaire-9 (PHQ-9), a tool previously validated in Spanish [45], and widely used in Peru [46]. Adolescents screening positive for mild or greater depressive symptoms (PHQ-9 ≥ 10) or with suicidal ideation were evaluated by Socios En Salud’s mental health program and offered Psychological First Aid [47] and referrals as needed. Participants with severe depressive symptoms (PHQ-9 ≥ 20) or suicidal ideation were offered linkage to free, specialized mental health services provided by the Peru Ministry of Health and delivered at nearby community mental health centers [48].

### Data collection

Data collection took place from October 2019 to January 2021 and included indicators of intervention feasibility and potential effectiveness, and social, clinical, and demographic factors that could challenge transition.

Feasibility indicators aligned with selected dimensions of acceptability and demand [49]. Acceptability focuses on the extent to which a program is judged as suitable or attractive. We assessed acceptability through study refusal rates (i.e., the proportion of potentially eligible individuals for the study who chose not to participate) and intervention retention rates (i.e., the proportion of individuals who were retained in the intervention). The demand domain describes the extent to which a new intervention is likely to be used. We assessed demand through intervention retention rates and group attendance for study activities. Group attendance was assessed by the proportion of individuals who attended at least one session, and the median number of groups attended by participants who attended at least one session.

Preliminary evidence of effectiveness is an impact indicator examining whether a new intervention shows promise of success with the intended population. We generated preliminary evidence of effectiveness by calculating changes in transition readiness at 9-months, relative to baseline, and in self-reported adherence, perceived instrumental and emotional support, self-efficacy and stress at 6, 9, and 12-months, relative to baseline. Follow-up measurements corresponded to the end of the intensive phase, the end of the taper phase, and three months after intervention, respectively.

*Baseline clinical data*, including CD4 cell count and viral load, were recorded from clinical charts. Follow-up measurements were rarely available due to testing disruptions resulting from the SARS-CoV-2 pandemic.

*Instruments.* Participants self-administered surveys in Spanish on a tablet device. We assessed self-reported adherence during the prior 30-day period with three questions: “How many days did you miss at least one dose of any of your ART medications?”; “How often did you take your ART medications correctly?” (5-point scale: always [= 5], almost always [= 4] sometimes [= 3], rarely [= 2], never [= 1]); “How well would you say you took your HIV medications, as directed by your doctor?” (6-point scale: excellent [= 6], very good [= 5], good [= 4], okay [= 3], bad [= 2], very bad [= 1]). We assessed perceived instrumental support, emotional support, perceived stress, and self-efficacy using the Spanish fixed-form version of the NIH Toolbox Emotion Battery (version 2.0) for ages 18–85. All have a 5-point ordinal scale ranging from “never” to “always”, with higher scores indicating higher levels [50, 51]. We evaluated transition readiness with two scales. “Am I On TRAC?” [52] consists of knowledge and behavior indices, which we adapted to the local context (see Text, Electronic Supplementary Materials 2, which describes the adaptations to the questionnaire). The knowledge scale assesses the respondent’s health condition and general medical self-care. The behavior scale measures the frequency with which respondents engage in individual health-related behaviors related to the developmental model of transition. We also used the Got Transition? checklist (version 2.0), which assesses personal health knowledge and use of medical services [53]. The questionnaire has a 3-point scale ranging from “not my responsibility” to “yes, I know this.” For both measures, we report the summed score of responses for each subscale; higher scores indicate greater knowledge, engagement or use.

### Statistical analysis

Feasibility indicators were reported as descriptive statistics. To examine changes in outcomes during the different phases of intervention implementation, we calculated frequencies, medians, and interquartiles ranges across time-points and calculated within-person change from baseline to each of the three follow-up points and tested whether this quantity differed from zero, using paired t-tests or Wilcoxon signed rank tests, as appropriate. For the NIH toolbox measurements, we report raw scores instead of the t-values validated in a U.S. population. To understand whether potential effectiveness differed among subgroups, we stratified analyses by early childhood versus recent diagnosis and whether the adolescent had been lost from care or had a history of chronic non-adherence. Data were analyzed using SAS version 9.4 (Cary, NC).

### Compliance with ethical standards

The authors declare no conflicts of interest. Ethical approval was granted in Peru by the Institutional Research Ethics Committees of the Instituto Nacional de Salud del Niño, Hospital Nacional Arzobispo Loayza, and Hospital Nacional Hipólito Unanue. In addition, ethical approval was granted by the Institutional Review Board (IRB) of Harvard Medical School in the USA. Written informed consent was obtained from adolescents 18 years of age and over or from guardian(s) of adolescents < 18 years. Participants < 18 years provided informed assent. Three adolescents < 18 years without a guardian provided assent and were granted a waiver of consent.

## Results

We enrolled 30 ALWH, with a median age of 19 years [IQR: 18–20] (Table I). The cohort included 19 (63%) ALWH who acquired HIV in early childhood, 13 (43%) ALWH who identified as female (including one transgender woman), 10 (33%) MSM, five (17%) Venezuelan nationals, and four young pregnant women (13%). Six (20%) had been lost from care at the time of enrollment. Of the 14 ALWH who transitioned from a pediatric clinic and were retained in treatment at enrollment, 10 (71%) had a suppressed viral load. The median number of self-reported days with at least one missed dose in the last 30 days was 2 [IQR: 0–13], and over half of participants reported taking their medications correctly “always” or “almost always” (score ≥ 4). Median emotional and instrumental support scores fell at the midpoint of the range of possible values while median self-efficacy and perceived stress fell below the midpoint of the range of possible values and median transition readiness fell at or above the midpoint of possible values (Table II).

**Table I:** Baseline characteristics of adolescent participants transitioning to adult HIV care, Lima, Peru

Characteristics	All participants N = 30 n(%)^a^	Early childhood infection N = 19 n(%)^a^	Recent infection N = 11 n(%)^a^
Age, in years, median [IQR]	19 [18–20]	19 [18–20]	19 [18–20]
Female assigned at birth	12 (40)	11 (58)	1 (9)
Female gender identity	13 (43)	11 (58)	2 (18)
Identifies as MSM	10 (33)	1 (5)	9 (82)
Pregnant	4 (13)	3 (16)	1 (9)
CD4 count cells/mm3 median [IQR]	544 [349–615]	553 [381–608]	535 [331–608]
Days from CD4 test to enrollment median [IQR]	130 [51–272]	235 [127–322]	55 [36–63]
Lost from care at the time of enrollment	6 (20)	6 (20)	0 (0)
Venezuelan nationality	5 (17)	1 (5)	4 (36)
At least one living parent	19 (63)	8 (42)	11 (100)
At least one traumatic life event^b^	21 (70)	11 (58)	10 (91)
Experienced sexual abuse (rape, attempted rape, or any type of forced or threatened sexual act)^b^	12 (40)	5 (26)	7 (64)
Urban hassles index median [IQR] (range 0–96)^c^	20 [13–27]	19 [11–27]	21 [17–27]
Unstable housing	4 (13)	4 (21)	0 (0)
Depression (mild to severe, N = 28)	16 (57)	10 (56)	6 (60)

^a^Unless otherwise noted

^b^Lifetime traumatic events were measured with the Life Events Checklist for DSM-5 (LEC-5) [54, 55]

^c^We assessed exposure to daily stressors using an adapted version of the 17-item Urban Hassles Index [55, 56], a tool designed to measure stressors affecting adolescents living in urban environments

**Table II:** Within-person changes in key outcomes among PASEO intervention participants (N = 30)

Outcome (range of possible values)	Score median [IQR]^a^				Within- person change from baseline median [IQR]^a^			P-value, within- person change from baseline^a^		
	**Baseline**	**6 mos.**	**9 mos.**	**12 mos.**	**6 mos.**	**9 mos.**	**12 mos.**	**6 mos.**	**9 mos.**	**12 mos.**
**Self-reported adherence to ART**										
Doses missed last 30 days, (0–30)^b,c^	2 [0, 13]	0.5 [0, 2]	1 [0, 3]	0.5 [0, 2]	0 [-11, 0]	-1 [-11, 0]	-1 [-12, 0]	0.03^d^	0.03^d^	0.01^d^
How often did you take your ART medications correctly, last 30 days? (1–5)^c,e^	4 [2, 5]	5 [4, 5]	4 [4, 5]	5 [4, 5]	1 [0, 2]	0 [0, 2]	1 [0, 2]	0.01^d^	0.01^d^	0.02^f^
How do you consider that you took your ART medications, as directed by your doctor, last 30 days? (1–6)^c,e^	4 [3, 4]	5 [4, 6]	4 [3, 5]	5 [4, 5]	1 [0, 2]^c^	0 [-1, 2.]	1 [0, 2]	0.001^d^	0.09^f^	0.02^f^
**Psychosocial outcomes**										
Emotional support (7–33)^e^	20 [13, 29]	22 [17, 31]	24.8 [16, 31]	25.8 [18.7, 32.7]	1 [-2, 5]	1 [-2, 5]	2 [-1, 10]	0.21^f^	0.10^d^	0.03^f^
Instrumental support (8–40)^e^	24 [16, 31]	26 [19, 31]	30 [21, 36]^g^	27.5 [24, 34]	0 [-3, 4.3]	2.6 [-1, 6]^g^	2 [0, 8]	0.67^d^	0.07^d,g^	0.004^d^
Self-efficacy (10–40)^e^	23 [19, 28]	25.5 [20, 30]	27 [21, 33]^g^	24.7 [21, 33]	1 [-4, 5]	3 [-2, 6]^g^	1.5 [-2, 8]	0.57^d^	0.03^f,g^	0.06^f^
Perceived stress (10–40)^b^	19 [17, 23]	19.2 [18, 22]	18 [15, 20]	18 [17, 20]	-0.5 [-3, 3]	-1.5 [-4, 2]	-1 [-5, 2]	0.59^f^	0.09^d^	0.04^f^
**Transition readiness** ^h^										
Got transition, my health (0–18)^e^	13.8 [12, 16]	--	16 [15, 18]	--	--	1.9 [0, 4]	--	--	< 0.001^f^	--
Got transition, health care usage (0–28)^e^	21 [18, 23.7]	--	24.1 [22, 27]	--	--	2.4 [1, 6]	--	--	< 0.001^f^	--
Am I ON TRAC, knowledge (12–60)^e^	48 [38.7, 52]	--	50.5 [47, 55]	--	--	3.3 [1, 8]	--	--	0.001^d^	--
Am I ON TRAC, behavior (9–45)^e^	28 [24, 31]	--	32 [27, 35]	--	--	3 [0, 6]	--	--	0.003^f^	--

^a^6, 9, and 12 months after enrollment correspond to the end of the intensive phase, the end of the taper phase, and three months after the intervention, respectively. ^b^Lower=favorable. ^c^ N=27 for baseline measurement and all within-person change comparisons. Three participants on ART for less than 7 days at the time of baseline data collection were excluded. ^d^ Wilcoxon signed rank. ^e^ Higher=favorable. ^f^ Paired T-test. ^g^ N=29. ^h^ Transition readiness was assessed at baseline and 9 months.

### Feasibility indicators

Of 36 adolescents referred for participation, six refused to participate (16.7%). Four were adolescents who had previously withdrawn from HIV care and did not wish to reengage. Two opted out because of scheduling conflicts with study activities. Once enrolled, no participant withdrew from the nine-month intervention, 12-month research study, or ART (12 months of follow-up). Of the 30 adolescents, all but three (90%) attended at least one in-person social support session. Based on timing of enrollment, 10 participants were eligible to have participated in five in-person social support sessions and 20 participants were eligible to have participated in three sessions. The median number of sessions attended by those participating in ≥ 1 session was two [IQR: 2, 3]). There were 41 virtual sessions offered, and 28 adolescents (93%) participated in at least one of these sessions (median number of sessions attended by those who participated = 34.5, [IQR:16.5–39.8]).

### Preliminary evidence of effectiveness

**Fig. 1:**
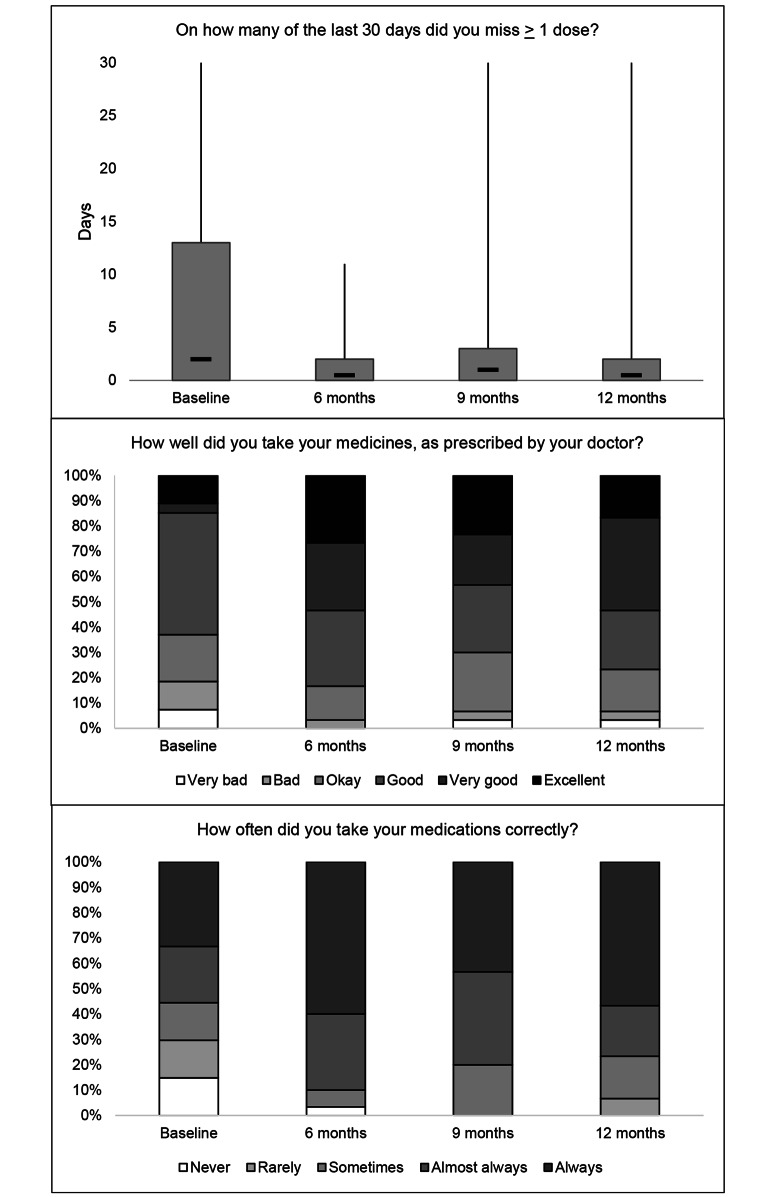
**Treatment adherence in the last 30 days among adolescents living with HIV in Lima, Peru.** In the graph of the number of days in the last 30 that at least one dose was missed, the bottom and top of the gray box represent the 25th and 75th percentiles, respectively. The dark horizontal like represents the median. The vertical error bars encompass the full range of values.

**Fig. 2:**
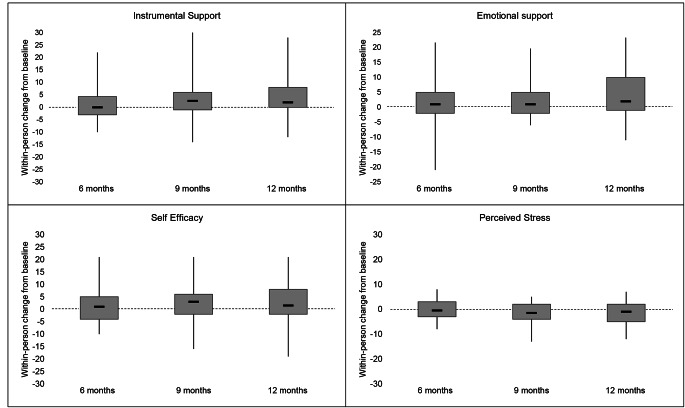
**Within-person changes in psychosocial outcome scores among adolescents living with HIV in Lima, Peru.** The bottom and top of the gray box represent the 25th and 75th percentiles, respectively. The dark horizontal line represents the median. The vertical error bars encompass the full range of values.

**Fig. 3:**
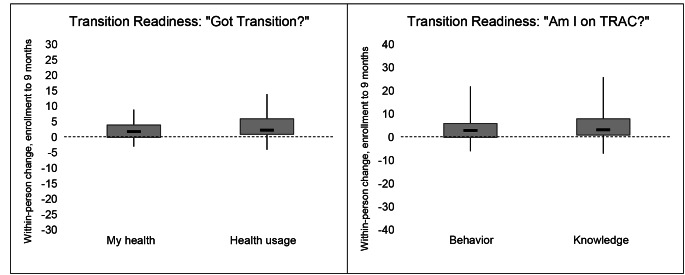
**Within-person changes in transition readiness among adolescents living with HIV in Lima, Peru**. The bottom and top of the gray box represent the 25th and 75th percentiles, respectively. The dark horizontal like represents the median. The vertical error bars encompass the full range of values.

Changes in adherence, psychosocial scale scores, and transition readiness scores are shown in Table II, Figs. 1, 2 and 3. In transition readiness, we observed within-person improvements related to personal health (+ 1.9 points, p < 0.001), healthcare usage (+ 2.4 points, p < 0.001), knowledge (+ 3.3 points, p = 0.001), and behavior (+ 3 points, p = 0.003) at the end of the intervention, relative to baseline (Table II). Results for adherence and psychosocial constructs followed a pattern of modest improvements in each scale between baseline and six-months, which increased further by nine-months and were generally sustained at twelve months (Table II). Although sample sizes were small for subgroup analyses, we observed evidence of improvements in transition readiness across all subgroups as well as improvements in adherence among adolescents living with HIV since early childhood, improvements in psychosocial indicators among adolescents with newly acquired HIV, and improvements in both metrics among adolescents with a history of non-adherence (Electronic Supplementary Materials 3-5).

## Discussion

We provide quantitative evidence supporting the feasibility and potential effectiveness of a community-based intervention designed to improve health outcomes for ALWH in Lima, Peru. The adolescents in this cohort were diverse in terms of their medical needs and social circumstances, which included depression, housing instability, poverty, lack of parental support, pregnancy and living in a foreign country. A key strength of the intervention was its comprehensive nature (i.e., it was designed to address a broad spectrum of unmet social and health needs) and individualized design (i.e., it sought to respond to each adolescent’s needs, tailoring the intensity of support offered to each participant). Research has highlighted the limited efficacy of a “one size fits all” approach for community-based interventions [10, 57, 58], in part because subgroups at highest risk may need extra support [30, 59]. In this intervention, entry-level health workers filled gaps in care, provided linkages, liaised with providers, and literally accompanied adolescents as they transitioned to adult care.

We found the PASEO intervention to be highly feasible based on metrics of acceptability and demand. There was a low refusal rate for enrollment, no participants were lost to follow up, and adolescents demonstrated high-attendance in group sessions. The potential effectiveness of PASEO is supported by within-person improvements in adherence, psychosocial indicators, and transition readiness; this improvement was often sustained three months after the intervention’s intensive phase. Importantly, these improvements appear to hold up across subgroups of adolescents characterized by timing of diagnosis (early childhood infection versus recent diagnosis) and among those with a history of non-adherence. This suggests that the lay health workers were effective at individualizing support to adequately meet the needs of each adolescent. Importantly, these data also corroborate that PASEO aligns with World Health Organization criteria for adolescent-friendly care in that it was acceptable (adolescents were willing to engage), equitable (all adolescents could access it), appropriate (offering the right menu of services) and effective (resulting in a positive contribution to health) [60]. To further increase equity, adaptations may be needed to ensure it is acceptable and effective for transgender women, as we only enrolled one in this study.

A number of intervention studies addressing the transition period are underway in low-or-middle-income and high-burden settings [61, 62]. While these studies will contribute importantly to knowledge on evidence-based practices, there is a notable lack of intervention studies addressing the transition to adult care in South America. This disparity reflects a broader knowledge gap related to effective interventions for ALWH in the Latin American and Caribbean. One recent systematic review of interventions for ALWH found only six studies that discussed linkage to care, none of which were from Latin America [63]; another, focused on psycho-social support interventions, identified six studies, but found none outside the U.S. or Africa. An intervention that was effective in improving continuity of care and psychosocial health outcomes among ALWH in South America would therefore contribute to filling an important research and implementation gap. And, the fact that the six adolescents who had been lost from care at enrollment remained engaged in ART at the end of the study, suggests that the PASEO intervention also may be an effective strategy for re-engaging ALWH who are lost from care.

This pilot study was underway when the SARS-CoV-2 pandemic emerged, during which stay-at-home orders were intermittently instated. These circumstances required us to adapt the intervention to a virtual format; however, improvements in key metrics suggest that the pillars of the intervention (accompaniment, social support, skills-building) were preserved under this hybrid model. The lack of a control group precludes definitively concluding that the intervention caused these improvements; however, baseline measurements preceded SARS-CoV-2, while follow-up measurements occurred in the first six months of the pandemic; therefore, the expected time trend in these outcomes would likely have attenuated any PASEO-related improvements [44]. While unplanned, the intervention adaptations could be considered a strength: the flexibility and high participation rates despite the pandemic suggest that the feasibility metrics are even more notable. And, successful adaptations open the possibility for flexible delivery across a variety of in-person parameters. The pandemic also resulted in temporary suspension of CD4 cell count and viral load testing, which prevented us assessing biologic endpoints. Finally, this analysis reports quantitative metrics of transition and well-being, and inferring clinical meaningfulness from the observed improvements is not straightforward. While within-person changes in scale scores for key constructs targeted by the intervention support its potential effectiveness, understanding participant and provider perspectives will be critical to its overall evaluation, as will identification of any differential effects across subgroups. Results from qualitative work with these groups will be reported.

## Conclusions

In conclusion, the PASEO intervention was feasible and demonstrated promise for improving myriad self-reported health outcomes in a diverse cohort of ALWH. Future studies should include a large-scale impact evaluation including biological outcomes and assessment of long-term effectiveness beyond the life of the intervention.

### Additional files

Additional file 1: Electronic Supplementary Materials.

Information on file format. Key components of intervention, overview of intervention implementation, adaptation of the “Am I on TRAC” transition readiness questionnaire, analyses of effectiveness outcomes, stratified by early childhood versus recent diagnosis and whether the adolescent had been lost from care or had a history of chronic non-adherence.

## Electronic supplementary material

Below is the link to the electronic supplementary material.

Supplementary Material 1

## Data Availability

Data can be provided upon request.

## List of abbreviations

ALWH: adolescents living with HIV.
ART: antiretroviral therapy.
DOT: directly observed treatment.
HIV: human immunodeficiency virus.
MSM: men who have sex with men.

## Code Availability

Data were analyzed using SAS version 9.4 (Cary, NC). Code can be provided upon request.
