# Supplementary material for: Community-based accompaniment for adolescents transitioning to adult HIV care in urban Peru: a pilot study
Source: AIDS Behav. 2022 Jul 5;26(12):3991–4003. doi: 10.1007/s10461-022-03725-2 (PMC9255463; doi:10.1007/s10461-022-03725-2)
Supplement: Supplementary file 1 — Supplementary Material 1 [file 10461_2022_3725_MOESM1_ESM.docx]

**Electronic Supplementary Materials**

**Electronic Supplementary Materials 1.**

| **Intervention activities** | **Months** | | | | | | | | | | |
| --- | --- | --- | --- | --- | --- | --- | --- | --- | --- | --- | --- |
|  | **0** | **1** | **2** | **3** | **4** | **5** | **6** | **7** | **8** | **9** | **10-12** |
|  | **Pre-transition** | **Post-transition** | | | | | | | | | **Follow-up** |
|  | **Intensive Phase** | | | | | | | **Taper phase** | | | **No intervention** |
| Social worker assessment^a^ | X |  |  |  |  |  |  |  |  |  |  |
| Depression screening and referral^a^ | X |  |  |  |  |  |  |  |  |  |  |
| Health insurance navigation, clinic accompaniment^a,b^ | X | X |  |  |  |  |  |  |  |  |  |
| In-person check-in with health promoter, once monthly^a^ → *virtual, weekly*^c^ | X | X | X | X | X | X | X | X |  | X |  |
| Clinic transportation stipend, as needed |  | X | X | X | X | X | X | X |  |  |  |
| Establish plan for taper phase |  |  |  |  |  | X | X |  |  |  |  |
| Social support groups, twice monthly → *replaced with virtual social activities* |  | X | X | X | X | X | X | X | X | X |  |
| Health education and skill-building sessions, monthly → *virtual, 1-3 times weekly* |  | X | X | X | X | X | X | X | X | X |  |
| **DOT, if indicated** |  |  |  |  |  |  |  |  |  |  |  |
| DOT daily → *virtual* |  | X | X | X | X | X | X |  |  |  |  |
| DOT twice weekly → *virtual* |  |  |  |  |  |  |  | X | X |  |  |
| DOT weekly → *virtual* |  |  |  |  |  |  |  |  |  | X |  |
| **Overview of PASEO intervention implementation.** DOT, Directly Observed Treatment   1. Occurred more frequently, as needed 2. Accompaniment to clinic visits typically occurred through months two or three, with longer durations for those previously lost from care, experienced adverse reactions or side effects of treatment, or had concomitant health issues. Clinic visit accompaniment occurred for shorter durations if the adolescent received this support from a caregiver or family member. 3. Italicized text represents modifications made in response to the SARS-CoV-2 pandemic | | | | | | | | | | | |

**Electronic Supplementary Materials 2. Adaptation of the “Am I on TRAC” transition readiness questionnaire.**

The 21-item questionnaire consists of a knowledge index and a behavior index. The original knowledge subscale consisted of 16 questions and four response categories, ranging from “strongly disagree” to “strongly agree”. However, based on feedback from the Youth Advisory Board, we added a “neutral” response item and removed four questions that were less applicable to the study population (“I know how to get my medical records”, “I know how my health condition might limit my career choices”, “I know how my health condition affects my physical activities”, and “I have a family doctor”).

**Electronic Supplementary Materials 3. Changes in key outcomes among PASEO intervention participants who were lost from care or had a history of chronic non-adherence to ART (N=13)**

| **Outcome (range of possible values)** | **Score**  **median [IQR]^a^** | | | | **Within- person change from baseline**  **median [IQR]^a^** | | | **P-value,**  **within- person change^a^** | | |
| --- | --- | --- | --- | --- | --- | --- | --- | --- | --- | --- |
|  | **Baseline** | **6 mos.** | **9 mos.** | **12 mos.** | **6 mos.** | **9 mos.** | **12 mos.** | **6 mos.** | **9 mos.** | **12 mos.** |
| **Self-reported adherence to ART** | | | | | | | | | | |
| Doses missed last 30 days, (0-30)^b^ | 5 [2, 30] | 0 [0, 2] | 1 [0, 3] | 0 [0, 2] | -5 [-29, 0] | -5 [-29, 0] | -3 [-30, -1] | 0.02^c^ | 0.02^c^ | 0.002^c^ |
| How often did you take your ART medications correctly, last 30 days? (1-5)^d^ | 3 [2, 4] | 5 [4, 5] | 4 [3, 5] | 5 [3, 5] | 2 [0, 3] | 1 [0, 3] | 1 [0, 2] | 0.02^c^ | 0.04^c^ | 0.04^c^ |
| How do you consider that you took your ART medications, as directed by your doctor, last 30 days? (1-6)^d^ | 3 [2, 4] | 4 [4, 5] | 5 [3, 5] | 5 [4, 5] | 1 [0, 3] | 1 [0, 3] | 1 [0, 2] | 0.02^e^ | 0.09^e^ | 0.008^c^ |
| **Psychosocial outcomes** | | | | | | | | | | |
| Emotional support (7-33)^d^ | 21 [13, 26] | 17 [12,28] | 18.7 [14,27] | 19 [18, 33] | 0 [-3, 5] | 1 [-2.3, 3] | 5 [-2.3, 8] | 0.84^e^ | 0.81^e^ | 0.24^e^ |
| Instrumental support (8-40)^d^ | 20 [15,26] | 20 [16,22] | 27.8 [16.5, 31] | 24 [20, 27] | 0 [-3, 4.3] | 3.5 [-1, 4.5] | 4 [2, 7] | 0.81^c^ | 0.09^e^ | 0.002^e^ |
| Self-efficacy (10-40)^d^ | 20 [19,24] | 21 [16,28] | 24 [21,30.5]^f^ | 21.1 [19, 26] | 1 [-3.7, 7] | 4 [-2.5, 10.5]^f^ | 2 [-2, 7] | 0.33^e^ | 0.07^e,f^ | 0.18^e^ |
| Perceived stress (10-40)^b^ | 20 [18,23] | 19.2 [18,22] | 19 [17,20] | 19 [17, 20] | 0 [-3, 3] | -1 [-2, 2] | -0.7 [-5, 2] | 0.82^e^ | 0.38^e^ | 0.17^e^ |
| **Transition readiness** ^g^ | | | | | | | | | | |
| Got transition, my health (0-18)^d^ | 14 [12,15] | - | 15.7 [15,18] | - | - | 1.2 [0.6, 3] | - | - | 0.01^e^ | - |
| Got transition, health care usage (0-28)^d^ | 22 [21,24] | - | 24.1 [23,28] | - | - | 2 [1, 5.1] | - | - | 0.001^c^ | - |
| Am I ON TRAC, knowledge (12-60)^d^ | 51 [46,52] | - | 54 [48,58] | - | - | 4 [2, 6.6] | - | - | <0.001^e^ | - |
| Am I ON TRAC, behavior (9-45)^d^ | 28 [24,29] | - | 28 [26,32] | - | - | 0 [-2, 6] | - | - | 0.29^e^ | - |

^a^6, 9, and 12 months after enrollment correspond to the end of the intensive phase, the end of the taper phase, and three months after the intervention, respectively

^b^Lower=favorable

^c^Wilcoxon signed rank

^d^Higher=favorable

^e^Paired T-test

^f^ N=12

^g^Transition readiness was assessed at baseline and 9 months

**Electronic Supplementary Materials 4. Changes in key outcomes among PASEO intervention participants with recent HIV diagnosis (N=11)**

| **Outcome (range of possible values)** | **Score**  **median [IQR]^a^** | | | | **Within- person change from baseline**  **median [IQR]^a^** | | | **P-value,**  **within- person change^a^** | | |
| --- | --- | --- | --- | --- | --- | --- | --- | --- | --- | --- |
|  | **Baseline** | **6 mos.** | **9 mos.** | **12 mos.** | **6 mos.** | **9 mos.** | **12 mos.** | **6 mos.** | **9 mos.** | **12 mos.** |
| **Self-reported adherence to ART** | | | | | | | | | | |
| Doses missed last 30 days, (0-30)^b,c^ | 0.5 [0, 5] | 1.5 [0, 3] | 1 [0, 2.5] | 1 [1, 3.5] | 0 [-4, 3] | 0 [-5, 2] | 0 [-3.5, 3] | 0.83^d^ | 1^e^ | 1^e^ |
| How often did you take your ART medications correctly, last 30 days? (1-5)^c,f^ | 5 [4, 5] | 5 [4, 5] | 4 [4, 5] | 5 [4, 5] | 0.5 [-0.5, 1] | 0 [-0.5, 0.5] | 0.5 [-1, 1] | 0.14^d^ | 0.80^d^ | 0.84^d^ |
| How do you consider that you took your ART medications, as directed by your doctor, last 30 days? (1-6)^c,f^ | 4.5 [4, 5] | 5 [4.5, 5] | 4.5 [4, 5] | 5 [4, 5] | 0.5 [0, 1.5] | 0 [-0.5, 1] | 0.5 [-1, 1] | 0.35^d^ | 1^d^ | 0.82^d^ |
| **Psychosocial outcomes** | | | | | | | | | | |
| Emotional support (7-33)^f^ | 18 [11, 30] | 26 [22, 31] | 29 [21, 31] | 31 [23, 32.7] | 1 [0, 13] | 2 [0, 18] | 3 [0.7, 20] | 0.05^e^ | 0.02^e^ | 0.04^e^ |
| Instrumental support (8-40)^f^ | 24 [16, 31] | 29 [25, 34] | 30 [25, 37] | 32 [27, 37] | 2 [-2, 12] | 5 [-1, 10] | 5 [2, 16] | 0.11^d^ | 0.11^d^ | 0.02^d^ |
| Self-efficacy (10-40)^f^ | 25 [19, 32] | 26 [24, 29] | 28 [20, 37] | 30 [24.4, 36] | 2 [-5, 5] | 2 [-2, 4] | 2 [-2, 14] | 0.45^d^ | 0.28^e^ | 0.05^d^ |
| Perceived stress (10-40)^b^ | 19 [17, 25] | 20 [17, 22] | 20 [15, 22] | 18 [18, 18] | -1 [-3, 3] | -1 [-4, 2] | -2 [-7, -1] | 0.46^d^ | 0.23^d^ | 0.02^d^ |
| **Transition readiness**^g^ | | | | | | | | | | |
| Got transition, my health (0-18)^f^ | 12 [10, 16] | - | 15 [14, 16] | - | - | 2.3 [-1, 6] | - | - | 0.09^d^ | - |
| Got transition, health care usage (0-28)^f^ | 19 [17, 23] | - | 23 [19, 27] | - | - | 2 [-0.5, 6] | - | - | 0.06^d^ | - |
| Am I ON TRAC, knowledge (12-60)^f^ | 43 [38, 49.1] | - | 50 [42, 52] | - | - | 3 [0, 8] | - | - | 0.07^d^ | - |
| Am I ON TRAC, behavior (9-45)^f^ | 28 [23, 29] | - | 34 [32, 38] | - | - | 6 [3, 9] | - | - | 0.008^d^ | - |

^a^ 6, 9, and 12 months after enrollment correspond to the end of the intensive phase, the end of the taper phase, and three months after the intervention, respectively

^b^ Lower=favorable

^c^ N=8 for baseline measurement and all within-person change comparisons. Three participants on ART for less than 7 days at the time of baseline data collection were excluded.

^d^ Paired T-test

^e^Wilcoxon signed rank

^f^ Higher=favorable

^g^ Transition readiness was assessed at baseline and 9 months

**Electronic Supplementary Materials 5. Changes in key outcomes among PASEO intervention participants with early childhood HIV infection (N=19)**

| **Outcome (range of possible values)** | **Score**  **median [IQR]^a^** | | | | **Within- person change from baseline**  **median [IQR]^a^** | | | **P-value,**  **within- person change^a^** | | |
| --- | --- | --- | --- | --- | --- | --- | --- | --- | --- | --- |
|  | **Baseline** | **6 mos.** | **9 mos.** | **12 mos.** | **6 mos.** | **9 mos.** | **12 mos.** | **6 mos.** | **9 mos.** | **12 mos.** |
| **Self-reported adherence to ART** | | | | | | | | | | |
| Doses missed last 30 days, (0-30)^b^ | 3 [0, 30] | 0 [0, 2] | 1 [0, 3] | 0 [0, 2] | -1 [-28, 0] | -1 [-17, 0] | -1 [-25, 0] | 0.02^c^ | 0.01^c^ | <0.001^c^ |
| How often did you take your ART medications correctly, last 30 days? (1-5)^d^ | 3 [2, 5] | 5 [4, 5] | 4 [4, 5] | 4 [3, 5] | 1 [0, 3] | 1 [0, 3] | 1 [0, 3] | 0.03^c^ | 0.006^c^ | 0.02^c^ |
| How do you consider that you took your ART medications, as directed by your doctor, last 30 days? (1-6)^d^ | 3 [2, 4] | 5 [4, 6] | 5 [3, 5] | 5 [4, 5] | 1 [0, 3] | 1 [-1, 3] | 1 [0, 2] | 0.004^e^ | 0.09^e^ | 0.002^c^ |
| **Psychosocial outcomes** | | | | | | | | | | |
| Emotional support (7-33)^d^ | 21 [13, 29] | 19 [13, 31] | 19 [14, 31] | 19 [17, 33] | 0 [-4, 5] | 0 [-2.3, 3] | 1 [-2.3, 8] | 0.99^e^ | 0.66^e^ | 0.24^e^ |
| Instrumental support (8-40)^d^ | 23 [15, 36] | 21 [17.3, 31] | 29.3 [19, 35]^f^ | 24 [20, 33] | 0 [-4, 1] | 1.8 [-2, 4]^f^ | 2 [-1.9, 7] | 0.47^e^ | 0.29^e,f^ | 0.21^e^ |
| Self-efficacy (10-40)^d^ | 23 [19, 26] | 22 [16, 31] | 24.5 [21, 31]^f^ | 23 [19, 28] | -1 [-3.7, 6] | 3 [-2, 9]^f^ | 0 [-2, 8] | 0.46^e^ | 0.09^e,f^ | 0.35^e^ |
| Perceived stress (10-40)^b^ | 19 [16, 23] | 19 [18, 22] | 17 [15, 20] | 18 [16, 21] | 0 [-4, 3] | -2 [-4, 2] | -0.7 [-5, 2] | 0.74^e^ | 0.12^e^ | 0.20^e^ |
| **Transition readiness**^g^ | | | | | | | | | | |
| Got transition, my health 0-18)^d^ | 14 [12.3, 16.8] | - | 17 [15, 18] | - | - | 1.7 [0.6, 3.7] | - | - | 0.001^e^ | - |
| Got transition, health care usage (0-28)^d^ | 22 [19, 24] | - | 25 [23, 28] | - | - | 2.7 [1, 6] | - | - | <0.001^e^ | - |
| Am I ON TRAC, knowledge (12-60)^d^ | 51 [46, 54] | - | 51 [48, 59] | - | - | 3.5 [1, 7] | - | - | 0.006^c^ | - |
| Am I ON TRAC, behavior (9-45)^d^ | 28 [24, 32] | - | 30 [26, 35] | - | - | 0 [-2, 6] | - | - | 0.16^e^ | - |

^a^ 6, 9, and 12 months after enrollment correspond to the end of the intensive phase, the end of the taper phase, and three months after the intervention, respectively

^b^ Lower=favorable

^c^ Wilcoxon signed rank

^d^Higher=favorable

^e^ Paired T-test

^f^ N=18

^g^ Transition readiness was assessed at baseline and 9 months
